# Nanoscale slip length prediction with machine learning tools

**DOI:** 10.1038/s41598-021-91885-x

**Published:** 2021-06-15

**Authors:** Filippos Sofos, Theodoros E. Karakasidis

**Affiliations:** Physics Department, University of Thessaly, 35100 Lamia, Greece

**Keywords:** Surfaces, interfaces and thin films, Information theory and computation

## Abstract

This work incorporates machine learning (ML) techniques, such as multivariate regression, the multi-layer perceptron, and random forest to predict the slip length at the nanoscale. Data points are collected both from our simulation data and data from the literature, and comprise Molecular Dynamics simulations of simple monoatomic, polar, and molecular liquids. Training and test points cover a wide range of input parameters which have been found to affect the slip length value, concerning dynamical and geometrical characteristics of the model, along with simulation parameters that constitute the simulation conditions. The aim of this work is to suggest an accurate and efficient procedure capable of reproducing physical properties, such as the slip length, acting parallel to simulation methods. Non-linear models, based on neural networks and decision trees, have been found to achieve better performance compared to linear regression methods. After the model is trained on representative simulation data, it is capable of accurately predicting the slip length values in regions between or in close proximity to the input data range, at the nanoscale. Results also reveal that, as channel dimensions increase, the slip length turns into a size-independent material property, affected mainly by wall roughness and wettability.

## Introduction

Fast fluid transport has been a broad field of investigation lately, evolving from a technological evolution that has allowed the commercialization of devices at the nanoscale, such as micro/nano-electro mechanical systems (MEMS/NEMS), lab-on-a-chip, and nanotubes with applications in water desalination, drug delivery, and functionalized material generation. To achieve high flux, the choice of channel material is pivotal. Following the graphene introduction and its potential application in fluidic systems^1^, carbon nanotubes (CNTs)^2^ and alternative configurations with boron nitride (BNNTs) and silicon carbide (SiCNTs)^3,4^, Black Phosphorus (BP) layers^5^, to mention a few, have emerged as promising means of fluid transport, at system sizes starting from 1 to 2 nm.

In contrast to the macroscale, at this scale confinement effects arise, with significant fluid ordering near the solid surface, non-constant viscosity values, and slip lengths that violate the continuum no-slip assumption^6,7^. It is now well-established that there exists a number of interfacial characteristics that affect fluid transport and the degree of slip. The strength of the fluid/solid interaction, thermal and geometrical wall roughness, wall and fluid densities, wall mass, temperature and pressure conditions are among these characteristics and have been thoroughly investigated in the literature^8–13^. It has been also shown that at low forcing, slip occurs due to the motion of a few particles which propagate along the wall/fluid boundary as a localized nonlinear mode, while, at high forcing, particles near the wall contribute equally to slip^14^. Nanobubbles have been also observed near hydrophobic surfaces^15^ and confirmed experimentally^16^, in a way that they form a layer that acts as a lubricant, significantly increasing the slip length.

In dealing with fluid flow inside nanochannels, one would face the question of which method to use to accurately calculate the slip length value. In experimental systems, the slip length can be extrapolated from the measured velocity profile inside the channel wall^17^ or from flow rate measurements^18^; nevertheless, experimental values differ significantly from simulation-extracted values^19^. For Couette and Poiseuiile flow, the slip length at the solid boundary, *L*_*s*_, is calculated from the linear Navier boundary condition as $L_{s} = {{u_{w} } \mathord{\left/ {\vphantom {{u_{w} } {\mathop {\left. {\frac{{du_{w,z} }}{dz}} \right|}\nolimits_{w} }}} \right. \kern-\nulldelimiterspace} {\mathop {\left. {\frac{{du_{w,z} }}{dz}} \right|}\nolimits_{w} }}$, where *u*_*w*_ the fluid velocity at the wall. This Non-equilibrium Molecular Dynamics (NEMD) method has to confront increased shear rates that could affect the accuracy of the result^20^. An alternative approach, based on Equilibrium Molecular Dynamics (EMD), has been also incorporated for slip length calculation under low shear rates by considering only the shear viscosity and the relaxation time, overcoming problems associated with NEMD methods^21,22^. However, contradicting results do exist and it has been found that the slip length may monotonically increase or decrease under the same conditions in water flows inside CNTs^23,24^.

Notwithstanding the richness of well-documented simulation and experimental methods being exploited for calculating material properties at the nano/micro-scale, machine learning (ML) statistical methods are currently gaining ground for replacing, under certain cirmumstances, classical physics-related procedures. Ιn this context, ML involves the calculation of parameters for a system designed to make decisions on unseen/missing data, based on data extracted from simulations, experiments or fetched from relevant databases^25^. ML techniques include, among others, artificial, convolutional and recurrent neural networks (ANN, CNN and RNN, respectively), simple, multivariate or kernel-ridge regression models, random forest, and tree-based methods^26–28^. These methods are based on data-mining from existing databases, usually enriched by new simulation or experimental data, and can be implemented only with a superficial understanding of the physical problem^29^. In the near future, it is expected that MD simulations will be used to extract training data for ML models, sigificantly reducing the computational cost required^30^ and may suggest a joint scheme across scales^31^.

Following the trend of exploiting the ample data selection from the literature, along with our simulation data, the aim of this work is to present an alternative method to tackle a widely investigated physical problem, the slip length calculation. ML techniques, such as multivariate regression (MVR), the multi-layer perceptron (MLP), and random forest (RF) are incorporated to train, test and predict the slip length at the nanoscale. Input data covers a broad range of parameters which have been computationally found to affect the degree of slip in flows at nanoslits, for fluids such as the Lennard–Jones (LJ) fluid, water, and methane at liquid state. Albeit far from replacing simulation methods that have matured over the years in classical physics, chemistry and engineering problems, we show next that ML techniques are capable of reproducing fast, accurate prediction of computationally intensive, and, sometimes, ambiguous properties, such as the slip length at the fluid/solid interface, where large temporal fluctuations have been observed^32^. Increased accuracy and efficiency is obtained by the MLP and RF methods, while MVR presents low performance, not managing to capture all non-linear effects involved in the calculations.

## Methods

### System model

A great part of the datasets used for training and testing the ML model comes from Lennard–Jones (LJ) simulations of a Poiseuille-like system, where a fluid (monoatomic liquid, water, methane) flows between two infinite solid walls (monoatomic wall, graphene, platinum, carbon), periodic in *x-* and *y-*directions (details given on the “Supplementary information”). Walls can be either smooth or grooved of various dimensions, and reflect several cases of wettability.

### Dataset

Slip length calculations are extracted from literature simulation data, along with data extracted from our MD model. To investigate the effect of various flow and channel parameters on slip length values, representative references are chosen that present the slip length as function of the channel width, wall/fluid wettability, wall/fluid particle atomic size, groove length and height, wall stiffness, system temperature, fluid density, and the external driving force^22,33–38^.

A number of 344 data points constitute the dataset. The dataset is divided in training points to feed the ML models and testing points to compare with predicted data, in a percentage of 80/20, respectively. After data collection, a normalization stage follows, to restrict the input value range, by removing the mean and scaling to unit variance

1 $$ \overline{x} = \frac{{x - x_{mean} }}{{x_{std} }} $$

Possible input correlations are investigated through the calculation of the Variation Inflation Factor, *V*

2 $$V=\frac{1}{1-{R}_{i}^{2}}$$

where ${\mathrm{R}}_{\mathrm{i}}^{2}$ the coefficient of determination for an independent variable. The threshold of ommitting an input is for *V* > 10.

Nonetheless, the input dimension here (nine inputs) is still high. The Principal Component Analysis (PCA) is a feature selection technique that could reduce the model dimensionality without affecting its performance. PCA incorporates the transformation of a data space to a feature space, in a way that the original data set can be represented by reduced points, while retaining most of its properties. Dimensionality reduction is achieved by discarding those input features that have small variances and retain only those terms that have large variances (detailed analysis can be found in the “Supplementary information”, and theoretical relations in^39^).

### Machine learning

Machine learning algorithms exploited are the statistical multiple (or, multivariate) linear regression (MVR), the multi-layer perceptron (MLP), and random forest (RF). Calculations and plots have been extracted with the Python language, using sci-kit learn^40^, statsmodels^41^, seaborn^42^ and Yellowbrick^43^ packages/libraries.

For a set of *n* independent input variables, the multi-variate regression model is described by

3 $$ Y = \sum\limits_{i = 1}^{n} {w_{i} X_{i} } + b $$

where, *w*_*1*_, *w*_*2*_,…,*w*_*n*_ are the regression coefficients that weight the impact of the respective *X*_*1*_, *X*_*2*_,…,*X*_*n*_ independent inputs on the dependent variable *Y* and *b* the bias term which equals the unknown error imposed in the model.

Artificial neural networks are widely incorporated when other statistical methods are not applicable. The basic element of an ANN is the perceptron. The learning process includes the adjustment of weighted connections between nodes until an efficient solution has been obtained. The multi-layer perceptron comprises internal layers between input and output nodes which increase the complexity of the model, though generally providing better statistics. Deviations of the predicted outputs from actual values are iteratively minimized by incorporating a backpropagation scheme^39^. The number of hidden layers (15 × 40 × 15) was determined by trial and error.

Random Forest is a decision tree method that considers an average prediction approach. Main characteristics of the method are the depth levels of the tree and the number of estimators (trees). Prediction is extracted from the output of multiple decision trees. Each decision node has the MSE value as criterior of splitting^44^.

## Results

### Data analysis

The ML algorithms inferred build a model from a number of inputs, they are trained by a percentage of the input data, follow a decision process and provide predictions, verified by the remaining part of the input data set. The choice of input parameters has been made on the assumption that they have an impact on slip length calculation. Input parameters shown in Table 1 (given in LJ reduced values^45^) are the channel width, *h*, the ratio of groove length to channel height, *h*_*l*_*/h*, the ratio of groove height to channel height, *h*_*d*_*/h*, the ratio of wall-to-fluid interaction ${\varepsilon }_{wf}/{\varepsilon }_{ff}$, the ratio of wall-to-fluid particle size ${\sigma }_{wf}/{\sigma }_{ff}$, the ratio of wall-to-fluid particle mass ${m}_{w}/{m}_{f}$, the external driving force, F^*^, the wall spring force constant, K^*^, the system temperature, T^*^, and the fluid density ρ^*^. The output is the slip length-to-channel width ratio, *L*_*s*_*/h.*

**Table 1:** Data set value range.

Input	*X*_*1*_	*X*_*2*_	*X*_*3*_	*X*_*4*_	*X*_*5*_	*X*_*6*_	*X*_*7*_	*X*_*8*_	*X*_*9*_	*X*_*10*_	*Y*
Parameter	${\varepsilon }_{wf}/{\varepsilon }_{ff}$	${\sigma }_{wf}/{\sigma }_{ff}$	${m}_{w}/{m}_{f}$	K*	F*	*h*_*l*_*/h*	*h*_*d*_*/h*	*h*	T*	ρ*	*L*_*s*_*/h*
Min	0.10	0.75	0.66	57.15	0.001	0.08	0.00	2.90	0.83	0.047	0.00
Max	2.24	2.52	20.00	1 × 10^4^	4.900	1.00	0.36	100.40	2.59	1.300	7.68

Data curation is an essential pre-processing stage in ML techniques, starting with data normalization in order to restrict the input value range^46^. To decide on possible multi-collinearity and exclude inputs from the ML models, reducing its order, the Variation Inflation Factor, *V*, is calculated for every input (calculations are shown in the “Supplementary information”). The conclusion drawn from the *V* values is that ${\sigma }_{wf}/{\sigma }_{ff}$ has to be removed from the model.

To further decrease model complexity, the dataset investigation has shown that the original 9-input data set can be transformed to a 6-input PCA model, without significant loss, as the cumulative proportion of the variance explained surpasses 90%. Details are presented in the “Supplementary information”.

### Multivariate linear regression

**Figure 1:**
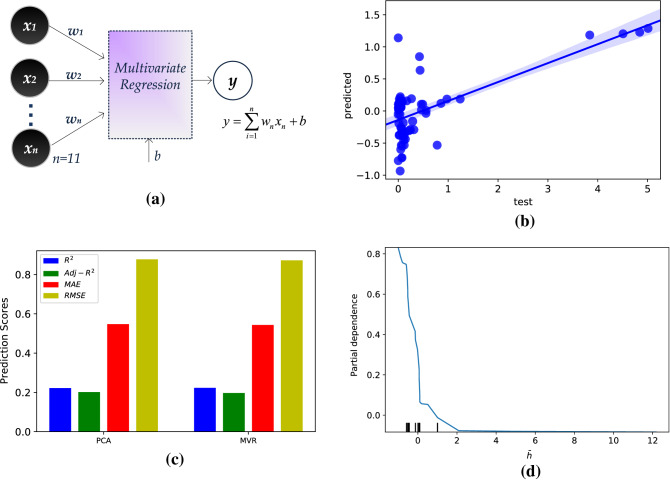
Model and results for the multivariate regression model (MVR), (**a**) data flow, (**b**) regression lines with 95% confidence level for the 9-input MVR model, (**c**) bar plot, quantifying R^2^, Adj-R^2^_,_ MAE, and RMSE for the MVR model and the PCA model, and (**d**) partial dependence plot, revealing the dependence on the (normalized) channel height, $\overline{h }$.

The MVR model exploits linear regression techniques to calculate the regression parameters *w*_*1*_–*w*_*n*_, so that the output (the slip length, *L*_*s*_*/h*) is extracted (Fig. 1a). After being trained, the MVR finds predictions and compares to the test data set. Regression lines with 95% confidence intervals are extracted for the MVR model in Fig. 1b, from which the uncertainty of predicted values over values used to test the model is quantified. The majority of test data points lie far from the regression line, indicating poor fitting. The PCA model has similar behavior to the 9-input MVR model (not shown here) and no accuracy loss is reported due to input feature reduction.

To further argue on the accuracy and the effectiveness of the MVR techniques exploited, the calculated prediction results (R^2^, Adj-R^2^_,_ MAE, RMSE) are presented in Fig. 1c. The MVR achieves R^2^ = 0.39, Adj-R^2^ = 0.20, MAE = 0.55, and RMSE = 0.88 while, for PCA, R^2^ = 0.36, Adj-R^2^ = 0.22, MSE = 0.54, and RMSE = 0.88. Although the obtained accuracy is low, the results validate the succesful PCA application for this model.

The partial dependence plot^47^ shown in Fig. 1d denotes the average marginal effect on the slip length prediction when the channel height, *h*, changes, while, in parallel, the other inputs remain unchangeable. The partial dependence is plotted on the vertical axis and $\overline{h }$(normalized *h*) on the horizontal axis. It is observed that for small values of $\overline{h }$ there is strong positive dependence which falls around − 0.1 for $\overline{h }\hspace{0.17em}$> 2 (which corresponds to the real value *h* $\cong$ 15 nm), meaning that the channel width no longer affects the slip length value. This finding presents macroscale behavior, similar to the classical no-slip assumption, since the slip length occurs only at the nanoscale and converges to zero when the channel dimension increases, as has been observed in relevant simulation studies^10,11^.

The linear MVR model seems to poorly approach slip length predictions at the nanoscale. We attribute this behavior to the fact that it is hard to find linear relations between input parameters and the calculated output, as every input affects the slip length in a different way. Channel geometrical characteristics and fluid/solid interactions affect the slip length the most, as stated in relevant works^22,33–38^. It has been shown that the slip length increases as the driving force increases, while it follows a fifth-order degree polynomial behavior when wall stiffness increases^33^. Moreover, slip length decreases with the presence of surface roughness^35^, when, at the same time, a hydrophilic surface amplifies this effect^48^.

### Multi-layer perceptron

**Figure 2:**
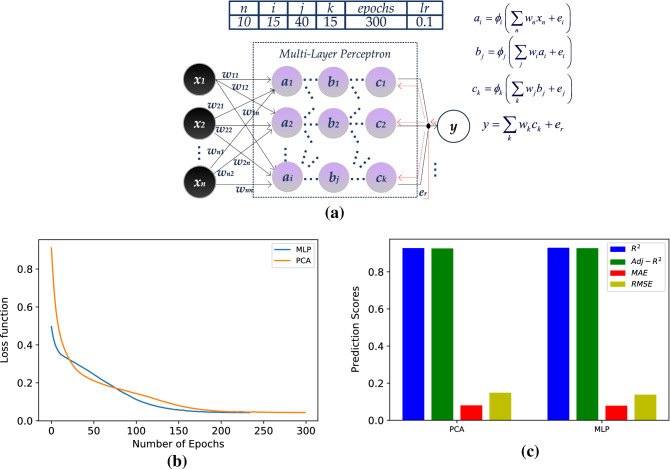
Model and results for the Multi-Layer Perceptron (MLR), (**a**) data flow, (**b**) loss functions, and (**c**) bar plot, quantifying R^2^, Adj-R^2^_,_ MAE, and RMSE for the MLP and the PCA models.

In a multi-layer perceptron (Fig. 2a), the weighted, training inputs move forward, towards the output, through the hidden (internal) layers. In every node, a rectified linear activation function (ReLU) is applied, which is common choice for the MLP. The obtained output is compared to the real data and an error signal is extracted. Adam optimizer and Mean Squared Error calculations for the loss function are considered in the MLP model. During the iterative backpropagation procedure, this error signal is propagated through the network and network weights are re-calculated, with a learning rate of *lr* = 0.01. It converges to its final value after several iterations (epochs) in which backward calculations from the output node to the MLP inner layers are held, until the error is minimized. The 9-input MLP (from now on, MLP) along with the PCA case are compared in Fig. 2b. The loss functions calculated converge in less than 200 epochs.

The bar plot in Fig. 2c depicts accuracy measurements for the MLP and the PCA cases exploited, in terms of R^2^, Adj-R^2^_,_ MAE, and RMSE. Compared to the respective quantities of the MVR technique, MLP clearly achieves better results, with R^2^ and Adj-R^2^ approaching 0.93, MAE = 0.08 and RMSE = 0.15. PCA input case seems to perform equally well to MLP.

### Random forest

**Figure 3:**
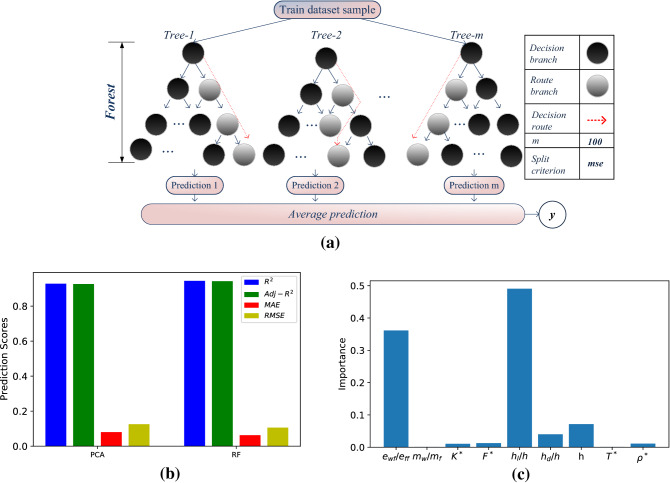
Model and results for the Random Forest (RF), (**a**) data flow, (**b**) bar plot, quantifying R^2^, Adj-R^2^_,_ MAE, and RMSE for the RF and the PCA models, (**c**) variable importance bar plot.

Random forests are characterized as effective prediction tools, that overcome overfitting issues^44^. They are usually incorporated for classification methods, but they can also achieve good performance scores in regression mode. In this model (Fig. 3a), each decision node (black squares) accepts the input parameters after a sequence of true/false decisions, and it concludes on the final slip length value. The predicted decisions are averaged in the end and the final slip length value is acquired. For the data set considered here, the RF accuracy measurements (Fig. 3b) show excellent performance on the original 9-input case (R^2^ = 0.94, Adj-R^2^ = 0.94, MAE = 0.06, and RMSE = 0.11), and similar for the PCA case (R^2^ = 0.93, Adj-R^2^ = 0.93, MAE = 0.8, and RMSE = 0.13). Figure 3c is a bar plot depicting the variable importance on the tree structure, e.g., it reveals the significance of a variable on the extracted prediction accuracy. Here we observe that results obtained verify simulation results; there is high importance from the roughness parameters, especially from the ratio *h*_*l*_*/h*, and the wettability strength ratio, ${\varepsilon }_{wf}/{\varepsilon }_{ff},$ in a percentage of 85%.

### Comparison of MVR, MLP, and RF methods

**Figure 4:**
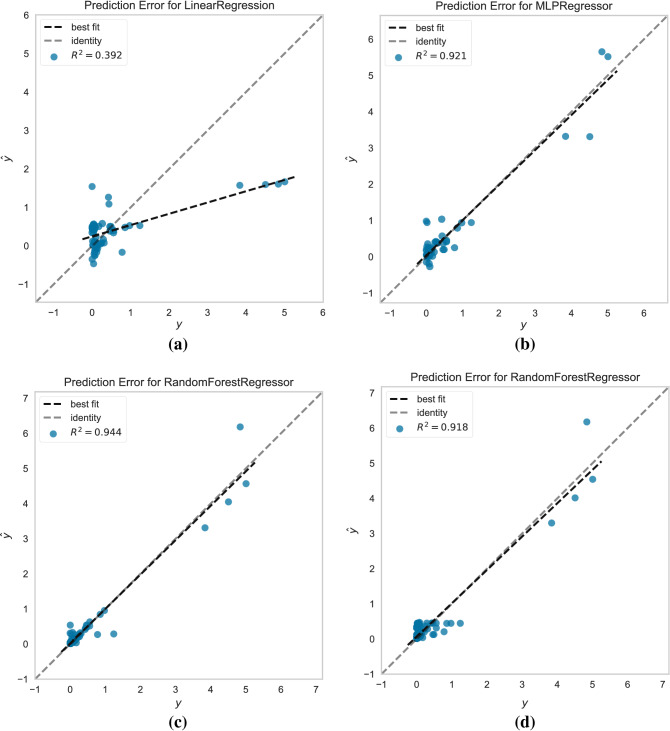
Prediction error plots for the (**a**) MVR, (**b**) MLP, (**c**) RF, and (**d**) RF-reduced, with only *h*_*l*_*/h* and ${\varepsilon }_{wf}/{\varepsilon }_{ff}$ as inputs. Grey dashed line is the 45° line and black dashed line the fitted line for each model.

Comparison of the MLP, MVR, and RF techniques incorporated in the previous sections is made with prediction error plots (Fig. 4a–c). This is a common ML scheme that presents the actual output versus the predicted values, revealing the model variance. A 45° degree line in the plots, denoting a perfect match between real and predicted values, is used for estimating how close the predictions approach model values. An almost perfect match is achieved for the MLP technique, where test points are close to the 45° regression line (Fig. 4b). On the other hand, there is low accuracy achieved by the MVR (Fig. 4a). However, the best score is achieved by the RF model in Fig. 4c, which generally fits well in physics-induced problems^49^. Following the variable importance result obtained from Fig. 4c, a prediction error plot for the RF method (RF-reduced) with only three input parameters, *h*_*l*_*/h*, *h*_*d*_*/h*, and ${\varepsilon }_{wf}/{\varepsilon }_{ff}$, is presented in Fig. 4d. It is of importance to note that prediction accuracy is only slightly reduced compared to the initial RF method.

**Figure 5:**
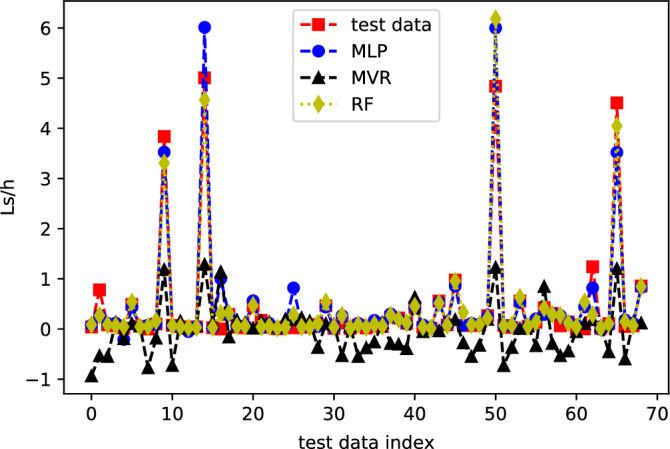
Comparison plot for test, MLP, MVR and RF data. Lines are guide to the eye.

The comparison between the three methods is further made clear on Fig. 5, where test and predicted data points are plotted together. MLP and RF values are in almost perfect match with test data, while, MVR values quantitatively follow the trend, but fail to incorporate the extreme values. It is also observed that the MVR model presents a great number of negative values for the slip length. This is due to extrapolation of linear functions that the MVR model suggests. It is, thus, obvious that linear regression models are not good choice when the dataset contains values around zero, with few extreme positive values (as in our case).

## Discussion

Adopting ML techniques in current physics and engineering problems is expected to broaden in all fields that deal with numerical data. The concept of predicting new, based on previous simulation, or experimentally extracted data, is constantly gaining ground. One of the properties of interest, as it affects material surface properties and the mass flow rate, is the slip length. The effect of slip length in channel fluid flows has been widely investigated in the context of nanofluidics. Surfaces that have been carefully engineered to attain specific properties are able to produce desirable slip lengths to control the flow rate in various applications.

In this work, having established a significant record of simulation data at the nanoscale with MD methods, along with high-quality data base records taken from the literature, we have turned our attention into employing ML statistical techniques to reveal the hidden behavior of slip at small scales. The proposed ML methods could override computationally demanding simulations, where possible, suggesting an alternative path between physics and statistics. The majority of available data comes from MD simulations. Care has been taken to select these datasets that comply to our simulation data. It is reported that there are several factors that affect the slip length, most of them as symptoms of confinement, such as channel wall wettability, roughness, shear rates, as well as flow characteristics such as density, temperature, and driving forces, to mention a few. Their effect on slip is sometimes contradicting; there are cases where, for example, the slip length increases until a maximum value when the channel height increases, while, there are cases where the opposite is observed. Moreover, although it has been observed that experimental and numerical data agree on slip lengths at the nanoscale, the experimental data are one order of magnitude larger^50^. From another point of view, it is noted that the slip length is purely a surface property and is not affected by the channel size, in water flows over carbon walls, for *h* > 5 nm^51^. The proposed ML methods here take into account the channel size and find that the slip length dependence reaches a plateau as *h* increases, suggesting that *h* affects the slip length only at small dimensions. From Fig. 1d, it is found that the slip length reaches the plateau value at around *h* > 15 nm and increases sharply for smaller *h* values.

A multi-parameter investigation has been established, where the slip length is extracted from several input data that have been previously found to control its behavior. Statistical tools have been exploited to replace the common simulation procedure, such as the Multi-Variate Regression, the Multi-Layer Perceptron, and Random Forest, three widely-applied ML techniques, capable of dealing with high-dimensional problems. A 9-input parameter vector is fed onto the models, which have one output, the slip length, from a set of 344 points. It has to be noted that the number of data points to incorporate is an open issue in ML techniques^52,53^. Nevertheless, the data set is representative of the problem that wishes to solve, e.g., it has incorporated data for a range of channel heights, wettability strengths, roughness characteristics (various combinations of height and length), wall spring constants, driving forces, fluid densities and temperatures.

A pre-processing stage is essential in order to constrain the input range values. A correlation check is also performed, to point out inputs that correlate to each other and may degrade the algorithm’s performance. It has been found that, for the specific data set, the atomic size ratio, ${\sigma }_{wf}/{\sigma }_{ff}$, presents strong correlation with the external driving force and must be removed from the calculations. This has led in a set of 9 input parameters. System dimensionality is still high; it becomes evident that when dealing with mass simulation data, one should keep dimensionality as low as possible. A feature selection technique, PCA, has been incorporated to further decrease the number of inputs. It has been shown that a 6-component PCA apply equally well in terms of accuracy on our data set, compared to the original 9-input, non-PCA case. In other words, the PCA method can be incorporated for supervised ML to diminish complexity and calculation time, and this would be a key issue in dealing with massive data.

Multivariate regression methods are first investigated on predicting slip length values. As the slip length is a result of confinement and classical theory is established on the no-slip assumption, it is expected that linear models would not fit well on slip length calculations. Researchers have found that, as a fluidic system reaches the microscale, slip lengths are reaching a plateau around zero, while there is significant slip at the nanoscale^54^. Furthermore, fluid properties concerning confinement effects (for example, the spring constant K^*^ or the solid/fluid interaction ${\varepsilon }_{wf}/{\varepsilon }_{ff}$) would deteriorate as system dimensions increase. We believe that MVR techniques could be successfully incorporated for channel flows in the region around 1–25*σ*, in the linear regime, without extreme shear rates, assisting current simulation methods in predicting fluid properties in cases where there are no simulation or experimental data. Having trained a ML model on carefully selected properties with a representative data set, one could predict values in-between the known points, reducing the computational effort and time needed by classical simulation methods.

The application of ML methods based on neural networks has shown remarkable performance on our data set. A Multi-Layer Perceptron with three hidden layers has been exploited, with forward and backward calculation capability, and seems to capture the effects of the input parameters on the slip length predictions, even in cases where the MVR model fails. Equally accurate results have been obtained with the Random Forest method. This model considers roughness and wall wettability significant in affecting the slip length values, verifying similar research efforts where hydrophobic walls were found to enhance fluid flux, similarly to nanobubbles or frictionless walls^55^. We remind the reader that the induced dataset includes fluid flows with LJ liquids, water, methane, over various wall materials (LJ, graphene, carbon, platinum) and structures (smooth, atomic, and geometrical roughness). This is promising result that could unveil fluid properties in even more complex fluidic configurations with ML methods. It has to be noted, nevertheless, that both MLP and RF methods are more computationally intensive, compared to MVR methods and this should be beard in mind in problems with huge datasets.

When simulation techniques are linked to statistical methods, the accurate system description is a matter of great importance. The fact that nine independent variables were chosen as the input parameter vector, has led into accurate predictions. However, there is always room for improvement. Increasing the training data set where possible is one choice. Moreover, better training would be made if data samples are laid in normal distance between them and they are not secluded in extreme values. Equally important is the choice of the ML algorithm to be used. Deep learning techniques are gaining in popularity in physics and chemistry applications nowadays^56^, having to anticipate for massive data, especially when dealing with first principles applications.

In the aftermath of the RF analysis, we note that another significant outcome has been obtained; the variable importance analysis has spotted only three parameters that affect slip length the most, at a percentage of 91.8%, the wall/fluid interaction ratio and the roughness parameters, *h*_*l*_ and *h*_*d*_. If this level of accuracy is acceptable, this result would disjoin slip length calculations from the channel dimension and make slip length a material property.

However, this finding is applied in a specific dimension range at the nanoscale. Further investigation has to be conducted towards this direction. To expand over a wider range, more simulation and/or experimental data is needed, while different wall materials and types of fluids have to be considered. All simulation data have been extracted under steady state conditions. To expand the model applicability in non-steady conditions, this would be a matter of future investigation. Moreover, training data would be necessary to incorporate on our ML model in order to draw predictions on multi-component fluid flow. Extracting experimental data and accurately record the experiment conditions is also a challenge. We believe that, if the system parameters are rigorously established, similar ML models would achieve high prediction scores and reveal the hidden dynamics of the processes inferred. There is a plethora of available ML algorithms that could be incorporated to construct a model able to provide predictions. Nevertheless, ML cannot be seen as a remedy that could replace all physics-based simulations, which have greatly matured over the years. It can be rather seen as a valuable tool that could provide missing/hidden properties among consecutive simulations, assist in scaling up and boost computationally intensive simulations.

To conclude, for efficient data mining, processing and prediction in physics, material science, chemistry, and engineering problems, machine learning can play a guiding role either as adjacent to simulation and experimental methods or as a promising future alternative. We draw attention on the importance of statistical methods in capturing the physical meaning of processes taking place at the nanoscale, where our ability to interfere is most of the times limited. Apart from making predictions, in a future work, we plan to employ more data science tools so as to suggest a general framework on discovering and approximating mathematical equations, which are expected to have wider applicability.

## Supplementary Information

Supplementary Information.

## Data Availability

Data and codes that support the findings of this study are available from the corresponding author upon reasonable request.
