# Supplementary material for: Nanoscale slip length prediction with machine learning tools
Source: Sci Rep. 2021 Jun 15;11:12520. doi: 10.1038/s41598-021-91885-x (PMC8206074; doi:10.1038/s41598-021-91885-x)
Supplement: Supplementary file 1 — Supplementary Information. [file 41598_2021_91885_MOESM1_ESM.docx]

**Nanoscale slip length prediction with machine learning tools**

Filippos Sofos^🖂^, Theodoros E. Karakasidis^🖂^

Physics Department, University of Thessaly, 35100 Lamia, Greece

^🖂^e-mail: fsofos@uth.gr; thkarak@uth.gr

**Supplementary information**

**Molecular Dynamics simulations.** The generation of datasets to be used for training and testing of the ML model come mainly from MD simulations, where the fluid of interest flows between two infinite solid walls. Data taken from the literature may have been obtained in different conditions, which are well-described in the respective papers. For the extraction of our own data, periodic boundary conditions are considered in *x*- and *y*-directions, while the distance between the walls is the channel height, *h*, consisting a Poiseuille-like flow system. The upper wall employs either a smooth surface or roughness elements, as shown in Figure S1.

The Lennard-Jones (LJ) 12-6 potential is applied

| $u_{LJ}=4\varepsilon\left[ \left( \frac{\sigma}{r_{ij}} \right)^{12}-\left( \frac{\sigma}{r_{ij}} \right)^{6} \right]$ | (S1) |
| --- | --- |

with parameters *σ* and *ε* the atomic size and potential, respectively. Other simulation parameters involved are the atomic mass, *m*, the fluid, *ρ_f_*, and wall, *ρ_w_*, density, and the cut-off radius, *r_c_*. Values of these parameters are different for every material under investigation. Examples are given in Table S1. The parameters *ε* and *σ* for two different species *α* and *b* are obtained by the Lorentz-Berthelot mixing rule $\sigma_{ab}=\left(\sigma_{a}+\sigma_{b}\right)/2$and$\varepsilon_{ab}=\sqrt{\left(\varepsilon_{a}\varepsilon_{b}\right)}$, based on the self-interaction parameters given in the Table S1.

For polar liquids, such as water, coulombic long-range interactions are further considered and given by$E=\frac{Cq_{i}q_{j}}{er}$, *r<rc*, where *C* is the energy conversion constant, *q_i_* and *q_j_* are charges of atoms and *e* the dielectric constant. The SPC/E (extended simple point charge) pair potential is chosen for our water model for its simplicity, computational speed, and efficiency. Water is expressed as a 3-site rigid molecule with charge *q*_H_ = 0.4238 for hydrogen and *q*_O_ = −0.8476 for oxygen. Water bonds are constrained using the SHAKE algorithm as implemented in LAMMPS [S1].

**Table S1.** Model parameters for various fluid and wall atomic pairs

| **Atom** | **σ(Å)** | **ε (Kcal/mol)** | **m (a.u.)** |
| --- | --- | --- | --- |
| Ar | 3.405 | 0.185 | 39.95 |
| Kr | 3.633 | 0.258 | 83.80 |
| H | 0.000 | 0.000 | 1.008 |
| O | 3.166 | 0.155 | 15.994 |
| C | 3.400 | 0.056 | 12.011 |


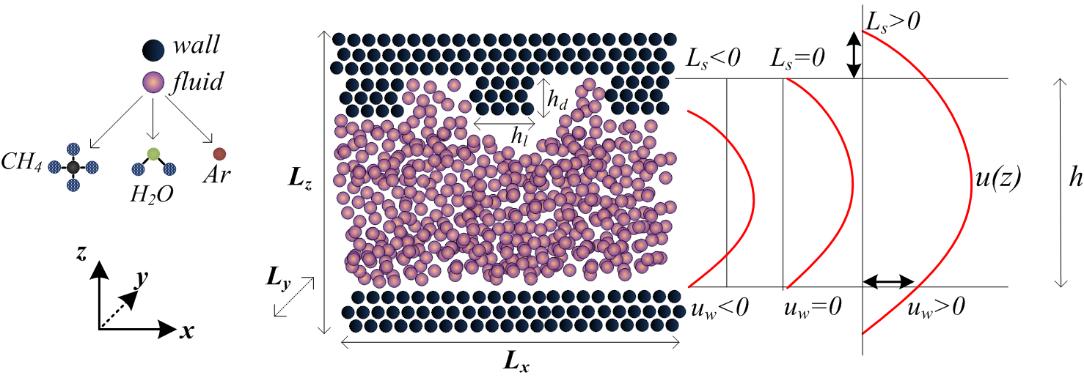


**Figure S1**. The general flow model. Red lines refer to velocity profiles, from which the slip length (ML data points) is extracted.

Wall particles are kept around their original position due to an elastic spring potential $u_{wall}(\left| r(t)-r_{eq} \right|)=\frac{1}{2}K(\left| r(t)-r_{eq} \right|)^{2}$, where *r(t)* is the position of a particleat time *t*, *r_eq_* is its initial lattice position and *K* is the spring constant. Large *K* values correspond to a rigid wall. After an initial NVE equilibration stage, a driving force $F_{ext}$ is applied to all fluid particles to generate flow, while system temperature *T* remains constant through the application of Nosé-Hoover thermostats in the NVT ensemble.

The slip length at the boundary, *L_s_*, is calculated from the linear Navier boundary condition as

| $L_{s}=u_{w}/\left.\frac{du_{w,z}}{dz}\right\vert_{w}$ | (S2) |
| --- | --- |

where *u_w_* the fluid velocity at the wall. The dimensionless slip length is given by the ratio *L_s_/h*. As shown in Fig. S1, it can be obtained by extrapolating the fluid velocity profile until it vanishes. A positive wall fluid velocity leads to positive slip. The no-slip condition appears when the slip length is zero, while a negative slip length denotes that the fluid velocity vanishes within the fluid before entering the wall. This may happen in case of surface roughness where the channel is practically narrowed^S9^.

Moreover, at the nanoscale, shear viscosity is affected by the presence of the walls and deviates from its bulk value. Next to a hydrophobic wall fluid atoms present smaller shear viscosity values, while, close to a hydrophilic wall, shear viscosity presents greater than the bulk values (a detailed description is given in ^S10^).

A summary of the data points incorporated for our ML model is given in Table S2. Channel dimensions *L_x_, L_y_, L_z_* are in reduced LJ units. The R/S column denotes the existence of a rough (R) or a smooth (S) wall, *N_f_* is the number of fluid particles during the simulation. The fluid and wall particle types are given, along with the total number of data points extracted from each reference source.

**Table S2: Data points from our own simulation data and data from the literature employed in our model.**

| ***L_x_(σ)*** | ***L_y_(σ)*** | ***L_z_(σ)*** | ***R/S*** | ***N_f_*** | ***Fluid type*** | ***Wall type*** | ***Data points*** | ***Source*** |
| --- | --- | --- | --- | --- | --- | --- | --- | --- |
| 10.62 | 10.62 | 4.43 - 50.22 | S | 320-1440 | Ar | Kr | 65 | [S2] |
| 10.62 | 10.62 | 4.43 - 50.22 | R | 648-1404 | LJ | LJ | 163 | [S2] |
| 8.81 | 8.81 | 4.41-8.81 | S | 891 | H2O (SPCe) | C | 7 | [S2] |
| 20.00 | 20.00 | 10.00 | S | 1728 | LJ | LJ | 27 | [S3,S4] |
| 16.97 | 6.53 | 32.91 | S | 2880 | LJ | LJ | 45 | [S5,S6] |
| 2.02-20.00 | 10.00 | 1.05-210 | S | 200-3032 | CH4 | C | 17 | [S7,S8] |
| 5.87 | 0.58 | 3.00 | R | 5200 | Ar | Pt | 20 | [S9] |

**Machine Learning metrics.** Error metrics used are the mean square error (MSE), the root mean square error (RMSE), the determination ocefficient, R^2^, and the adjusted determination coefficient $R_{\mathrm{adj}}^{2}$

| $MSE=\frac{1}{n}\sum_{i=1}^{n} \left.\left\vert Y_{i}-\hat{Y}_{i}\right.\right\vert$ | (S3) |
| --- | --- |

| $RMSE=\sqrt{\frac{1}{n}\sum_{i=1}^{n} \left(Y_{i}-\hat{Y}_{i}\right)^{2}}$ | (S4) |
| --- | --- |

The $R_{\mathrm{adj}}^{2}$ coefficient is a modified measure of the R^2^ coefficient, which integrates the significance of the independent variables, considering their number in the model. In a dataset with *n* points, with *k* independent variables, the adjusted R^2^ is given by

| $R_{adj}^{2}=1-\left[ \frac{\left( 1-R^{2} \right)\left( n-1 \right)}{n-k-1} \right]$ | (S5) |
| --- | --- |

The Variation Inflation Factor, *V*, is calculated for every input and values are tabulated in Table S3. The conclusion drawn from the *V* values is that ${\sigma_{wf}}/{\sigma_{ff}}$ has to be removed from the model. After the exclusion of this input, *V* values are re-calculated and no further collinearity is observed, since *V’* is below the threshold of 10 for every remaining input.

**Table S3:** Variation Inflation factor, before (*V*) and after (*V’*) the exclusion of ${\sigma_{wf}}/{\sigma_{ff}}$. $R_{i}^{2}$is the coefficient of determination for an independent variable

|  | ${\varepsilon_{wf}}/{\varepsilon_{ff}}$ | ${\sigma_{wf}}/{\sigma_{ff}}$ | ${m_{w}}/{m_{f}}$ | *K*^*^ | *F^*^* | *h_l_/h* | *h_d_/h* | *h* | *T^*^* | *ρ^*^* |
| --- | --- | --- | --- | --- | --- | --- | --- | --- | --- | --- |
| $V=\frac{1}{1-R_{i}^{2}}$ | 1.098 | 12.470 | 1.530 | 3.699 | 8.041 | 1.584 | 1.341 | 1.231 | 6.518 | 2.550 |
| $V'=\frac{1}{1-R_{i}^{2}}$ | 1.093 | - | 1.530 | 3.257 | 3.378 | 1.583 | 1.330 | 1.231 | 4.696 | 2.074 |

**Principal Component Analysis.** In systems where high dimensionality exists, PCA is employed to approximate the variation in *p* prediction variables using *k<p* transformed components. The result is smaller number of input variables that still explain most of the data variance.

The slip length data set incorporated here, after the application of a PCA algorithm, is transformed to a 9-component set. Component values are shown in Table S4. Every *PCA_i_* component (*i*=1-9) is given by

| ${PCA}_{i}=\sum_{j=1}^{9} w_{j}\times x_{j}$ | (S6) |
| --- | --- |

In order to select the optimal number of components, the scree plot method is chosen. The scree plot presents the number of components vs. the proportion of the variance explained. By defining a threshold, e.g., a percentage of the variance that should be followed by the model, the number of components is selected, and the components of little variance are discarded. In Figure S2, the first 6 out of the total 9 components are selected since they achieve 90.2% variance.

**Table S4.** PCA components. Shaded cells depict the most important input on each PCA parameter.

| **IN** | **property** | **PCA1** | **PCA2** | **PCA3** | **PCA4** | **PCA5** | **PCA6** | **PCA7** | **PCA8** | **PCA9** | **weight** |
| --- | --- | --- | --- | --- | --- | --- | --- | --- | --- | --- | --- |
| **x1** | ${\varepsilon_{wf}}/{\varepsilon_{ff}}$ | -0,007 | -0,064 | 0,483 | 0,232 | 0,455 | -0,316 | 0,314 | 0,532 | -0,144 | ***w1*** |
| **x2** | ${m_{w}}/{m_{f}}$ | 0,254 | -0,124 | -0,238 | 0,667 | -0,049 | 0,028 | -0,275 | 0,273 | 0,514 | ***w2*** |
| **x3** | *K*^*^ | -0,410 | 0,698 | 0,142 | 0,066 | -0,051 | -0,093 | 0,214 | -0,080 | 0,507 | ***w3*** |
| **x4** | *F^*^* | -0,350 | -0,125 | 0,350 | 0,081 | -0,438 | 0,632 | -0,020 | 0,367 | -0,082 | ***w4*** |
| **x5** | *h_l_/h* | 0,614 | 0,161 | -0,024 | -0,010 | -0,040 | 0,408 | 0,649 | -0,032 | 0,075 | ***w5*** |
| **x6** | *h_d_/h* | -0,510 | -0,420 | -0,432 | 0,153 | 0,274 | 0,149 | 0,479 | -0,134 | 0,098 | ***w6*** |
| **x7** | *h* | -0,027 | 0,361 | -0,142 | 0,021 | 0,658 | 0,522 | -0,334 | 0,061 | -0,168 | ***w7*** |
| **x8** | *T^*^* | 0,077 | -0,364 | 0,336 | -0,483 | 0,279 | 0,144 | -0,126 | -0,045 | 0,632 | ***w8*** |
| **x9** | *ρ^*^* | -0,019 | 0,116 | -0,498 | -0,482 | -0,093 | -0,103 | 0,046 | 0,690 | 0,088 | ***w9*** |


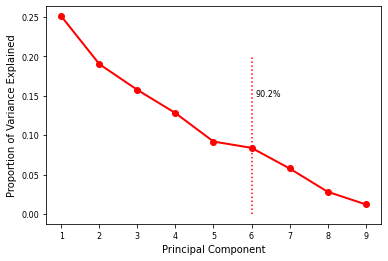


**Figure S2.** Scree plot, indicating that by incorporating 6 PCA inputs instead of the original 9, our model can achieve up to 90.2% of the input variance.

**References**

1. Plimpton, S. Fast parallel algorithms for short-range molecular dynamics. *J. Comput. Phys*. **117**, 1–19 (1995).
2. *Data extracted from our own simulations.*
3. Yang, S.C., Fang, L.B. Effect of surface roughness on slip flows in hydrophobic and hydrophilic microchannels by molecular dynamics simulation. *Molecul. Simulat* **31**(14-15), 971-977 (2005).
4. Yang, S.C. Effects of surface roughness and interface wettability on nanoscale flow in a nanochannel. *Microfluid. Nanofluid*. **2**, 501–511 (2006).
5. Asproulis, N., Drikakis, D. Boundary slip dependency on surface stiffness. *Phys. Rev. E* **81**, 061503 (2010).
6. Asproulis, N., Drikakis, D. Wall-mass effects on hydrodynamic boundary slip. *Phys. Rev. E* **84**, 031504 (2011).
7. Sokhan, V.P., Quirke, N. Interfacial friction and collective diffusion in nanopores. *Molecul. Simulat.* **30**(4), 217-224 (2004).
8. Sokhan, V.P., Quirke, N. Slip coefficient in nanoscale pore flow. *Phys. Rev. E* **78**, 015301 (2008).
9. Cao, B.Y., Chen, M. Guo, Z.Y. Liquid flow in surface-nanostructured channels studied by molecular dynamics simulation. *Phys. Rev. E* **74**, 066311 (2006).
10. Liakopoulos, A., Sofos, F., Karakasidis, T.Ε. Darcy-Weisbach friction factor at the nanoscale: From atomistic calculations to continuum models. *Phys. Fluids.* **29,** 052003 (2017).
